# Automated Recognition of Epileptic EEG States Using a Combination of Symlet Wavelet Processing, Gradient Boosting Machine, and Grid Search Optimizer

**DOI:** 10.3390/s19020219

**Published:** 2019-01-09

**Authors:** Xiashuang Wang, Guanghong Gong, Ni Li

**Affiliations:** 1 State Key Laboratory of Virtual Reality Technology and Systems, Beihang University, Beijing 100191, China; wxs_sky@outlook.com; 2 Automation Science and Electrical Engineering, Beihang University, Beijing 100191, China; ggh@buaa.edu.cn

**Keywords:** recognition of epilepsy EEG, Symlet wavelet, gradient boosting machine, grid search optimizer, multiple performance indices evaluation

## Abstract

Automatic recognition methods for non-stationary electroencephalogram (EEG) data collected from EEG sensors play an essential role in neurological detection. The integrated approaches proposed in this study consist of Symlet wavelet processing, a gradient boosting machine, and a grid search optimizer for a three-class classification scheme for normal subjects, intermittent epilepsy, and continuous epilepsy. Fourth-order Symlet wavelets are adopted to decompose the EEG data into five frequencies sub-bands, such as gamma, beta, alpha, theta, and delta, whose statistical features were computed and used as classification features. The grid search optimizer is used to automatically find the optimal parameters for training the classifier. The classification accuracy of the gradient boosting machine was compared with that of a conventional support vector machine and a random forest classifier constructed according to previous descriptions. Multiple performance indices were used to evaluate the proposed classification scheme, which provided better classification accuracy and detection effectiveness than has been recently reported in other studies on three-class classification of EEG data.

## 1. Introduction

Epilepsy is one of the most common neurological disorders, with one person in every 100 worldwide suffering from this disease [1]. Epileptic episodes are a clinical manifestation of paroxysmal abnormal ultra-synchronized electrical activity in the brain, which is iterative, sudden, and temporary. The automated detection of an epileptic episode and subsequent alert can aid neurologists who are monitoring treatment in busy neurological wards, and could help to ensure patient safety [2]. However, the time frequencies of epileptic episodes are uncertain, and their clinical manifestations are not easy to detect.

In an early system for testing and monitoring patients for epilepsy, researchers attempted to use sensors [3] to collect biological data from the patient’s surface, including electrocardiogram (ECG), electromyography (EMG) [4,5], motion data [6], and electrodermography (EDG). These data can be collected by wearable systems, including E-textiles [7]; capacitive sensing [8]; polymer materials, such as carbon nanotube (CNT)-polydimethylsiloxane (PDMS) [9]; Ag/AgCl electrodes [10]; and micro-needle arrays [11]. Wearable sensor systems can non-invasively monitor the biological signals of epileptic patients for long periods. However, in contrast to these types of biological data, brain signals can directly provide more information about changes in the brain.

Therefore, approaches to directly obtain epilepsy information from the brain have been researched, including positron emission tomography (PET), single photon emission computed tomography (SPECT), magnetic resonance imaging (MRI), and functional magnetic resonance imaging (fMRI) [12]. Currently, most studies focus on the use of video-electroencephalograms (EEGs) [13,14]. EEGs not only display temporal information, but also provide spatial information on electrical activity in the brain. The video-EEG technique has been considered a gold standard tool for the study of epilepsy. The real time information on an epileptic episode reflected by EEG cannot currently be replaced by any other physiological brain function monitoring method. As the physiological processes of seizure are typically non-stationary, dynamic, and nonlinear, the differentiation of rhythmic discharges from nonstationary processes brings great challenges to the analysis of EEG signals.

In general, the automated detection of EEG signals includes the two core tasks of feature extraction and classification. The extracted features can be divided into four categories: Statistical features, fractal dimension features, entropy features, and time-frequency domain features. Several studies have used combined time and frequency features for the automatic recognition of non-stationary EEG at the onset of epilepsy. Gotman was a pioneer in the exploration of automatic seizure detection technology based on EEG, and to capture transient behavior during long-term EEG monitoring, he decomposed the EEG into half waves and recorded the typical peak value as a morphological feature [15]. A classic example is the Welch spectral analysis method introduced into the feature analysis of epileptic seizure detection. Tzallas et al. used different time-frequency domain methods to extract the power spectrum density of computerized EEG signals during epileptic seizures [16]. Independent components analysis (ICA) [17] and linear discriminant analysis have also been reported for EEG signal extraction, while a multiscale radial basis function algorithm recently showed promising results in the decoding of EEG of epileptic seizures [18]. After consideration of the above-mentioned literature, Polat et al. proposed a hybrid model for seizure detection using a fast Fourier transform (FFT) for feature extraction [19]. To a certain degree, the most commonly used FFT reflects the frequency characteristics of the entire signal. Using FFT can process smooth and slow-changing signals over time. However, the FFT has several disadvantages: It can only show the overall behavior of the signals, and it cannot reflect the frequency variations of non-stationary EEGs using a fixed window function. Therefore, the short-time Fourier transform (STFT) was used to extract the frequency features from the raw EEG recording. The STFT algorithm performed time-frequency analysis of non-stationary EEG signals by adjusting different time windows to avoid the disadvantages of the FFT [20,21]. The original signals were truncated into smaller sections and windowed, and the discrete Fourier transform was applied to the signals. Under the assumption that the windowed signals represent stationary signals in different finite time widths, the power spectrum at different time periods can be calculated. The STFT treated non-stationary EEG as stationary signals and superimposed a series of short signals. In another approach, Boashash et al. extracted statistical and image features according to their time-frequency distribution to handle multichannel EEG from neonates [22]. A sensitivity of the criterion is that it is taken into consideration in the feature selection, resulting in a reduction in computational cost and improvement in detection performance [23]. Flexible wavelet transforms and the fractal dimension of the time-frequency method were also used for the detection of seizure segments in long-term EEG [24,25,26,27,28,29]. It is an analysis method that combines both the time and frequency domains. The wavelet transform decomposes the signal into different frequency bands, and expresses the nature and characteristics of the signal according to the “wavelet family” in these different frequency bands. The wavelet transform generally performs better than the FFT and STFT without prior knowledge. Discrete wavelet transforms (DWTs) have the ability to capture the frequency information in epileptic EEG, and many researchers have used Daubechies wavelets to analyze epileptic EEG signals, as they considered the Daubechies-4 wavelets to be similar to the spike wave of the EEG signals. The above literature indicates that the wavelet transform is the most commonly used method for extracting EEG features, although this extraction method ignores the overall statistical information. Therefore, we aimed to find an EEG analysis method that combines frequency information and statistical information.

For the automatic detection of EEG by machine learning, most studies have adopted a supervised learning paradigm. Regardless of the category of the input EEGs, the EEGs used to train classifiers are labeled according to prior knowledge. He et al.’s neural network (NN) classification technique used machine learning applied in the field of brain science [30]. Boser et al. [31], Fu et al. [32], and Gue et al. [14] all used support vector machines (SVMs) to identify the EEG signals of epilepsy patients, and obtained a relatively good recognition performance. Brabanter et al. proposed a least squares support vector machine (LS-SVM) to classify two-class seizure and non-seizure EEG signals from the small seizure dataset of Bonn University. They obtained 98.0–99.5% accuracy using a radial basis function (RBF) kernel, and 99.5–100% accuracy using a Morlet kernel [33]. Sun et al. used an Ada-Boost classifier to achieve good accuracy for spike detection of epileptic seizures [34]. However, the choice of a suitable strategy for machine learning is a difficult one, and numerous classification strategies have been developed for seizure detection, including random forests (RF), K-nearest neighbors (KNN) [35], and Bayesian neural networks [36]. The classification results indicate that these pattern recognition systems can achieve high levels of classification accuracy, from 93% to 99.66%. Nevertheless, these accuracy scores are the results of two-class EEGs classification, and the above mentioned schemes are relatively inconvenient and time consuming for practical clinical applications. Recently, Wang et al. explored a three-class classification problem, analyzing continuous ictal epilepsy patients, intermittent epilepsy patients, and healthy subjects. Using an SVM-based recognition system, they achieved an accuracy of 93.9% for the Bonn datasets [37]. However, the optimizer is not used to optimize the parameters in the process of training the model. It only relies on its own experience to adjust the parameters of the classified model. Therefore, a more effective classification scheme needs to be developed to solve the multi-class classification problem presented in this work.

After completing the feature extraction and classification procedures, it is also essential to perform a reasonable assessment to verify their accuracy. In the studies of machine learning for the assessment of EEG, the pursuit of high classification accuracy in a recognition system does not satisfy comprehensive assessment of the classification performance of the classifiers. Additional performance metrics revealing the causes of error in classification are also important in epilepsy detection. Recognition systems achieving high levels in these verification indicators could help fill gaps in the analysis of seizure monitoring devices, and reduce the rate of missed detections in clinical situations [38]. The Mayo Clinic and University of Pennsylvania hosted a competition to find robust seizure detection systems in 2014. Participants used SVM and random forest (RF) machine learning techniques on canine and human cortical electroencephalogram (ECoGs) datasets, and gained high sensitivity and low false-positive rates [39,40].

The remainder of the paper is organized as follows. In Section 2, we introduce a process flow of the proposed scheme for seizure EEG detection. In Section 3, we apply time-frequency and statistical methods to real EEG data after first preprocessing it. This study adopts the principal component analysis (PCA) method to reduce the dimensionality of the EEG features. We build the novel automatic gradient boosting machine (GBM) recognition system using 10-fold cross-validation (CV). In Section 4, we apply the automatic detection method to real EEG data to classify the three categories of seizure, light-seizure, and non-seizure EEGs to verify the effectiveness of the machine learning system. We compared and analyzed the performance of the GBM, SVM, and RF classifiers. The experimental results are analyzed using accuracy, confusion matric (CM), precision–recall curve (PRC), receiver operating characteristic (ROC), and the area under the curve (AUC) generated from the sensitivity and specificity. At last, the contributions of this research and future work are summarized in Section 5.

## 2. Proposed Scheme for Seizure EEG Detection

**Figure 1:**
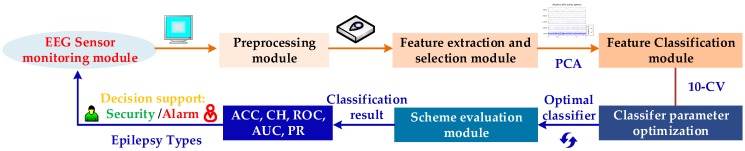
Auxiliary medical diagnostic system for epilepsy electroencephalogram.

The automatic integrated epileptic seizure EEG classifier proposed in this paper comprises five major modules, as illustrated in Figure 1. In the first step, signals are collected from EEG sensors in the monitoring module. In the second step, the data are entered in the preprocessing module for denoising. In the next step, a wavelet transform is adopted to analyze the time-frequency information of epilepsy, which avoids the shortcomings of the Fourier, STFT, and Welch spectral analyses. The Symlet wavelet is used to decompose the EEG signals into the γ, β, α, θ, and δ sub-bands. Then, the statistical information of the five sub-bands is extracted to generate the features for the feature extraction and selection module. PCA is applied to reduce the feature dimensionality, which is beneficial with respect to the computer runtime. Most previous studies have applied recognition algorithms to the Bonn epilepsy dataset, which is classified into seizure EEG epochs and non-seizure EEG epochs for two-class classification. Such two-class classification schemes are not ideal for practical applications because, in reality, there are multiple degrees of epileptic seizures. To obtain an efficient three-class classification scheme, we propose a GBM to classify the dataset. Labeled testing data are fed into the feature classification module. Then, a grid search optimizer (GSO) is performed to search for the best hyper-parameter values and to optimize the recognition system. We also implemented two other state-of-the-art machine learning classifiers, a support vector machine (SVM) and a random forest (RF), and compared them with the GBM classifiers. Finally, we used multiple performance indices to evaluate the scheme, and an evaluation module to detect the performance of the classifier. The comparison demonstrates that the GBM classifier is the most effective for identifying epileptic-state EEG. This recognition scheme not only ran faster than the SVM and RF, but also effectively avoided the misdiagnoses or missed diagnoses caused by the manual tuning of parameters. Our auxiliary medical diagnostic system can directly recognize three classifications from epilepsy EEG signals: Continuous ictal epilepsy patients, intermittent epilepsy patients, and healthy subjects.

## 3. Scheme Implementation

### 3.1. Real Epilepsy EEG Dataset

This study used an open-source database available from the University of Bonn to extract the key features for detecting continuous ictal epilepsy patients, intermittent epilepsy patients, or healthy volunteers using EEG signals [41,42]. These datasets have been widely used to test many methods and can be considered as a benchmark for developing seizure detection schemes.

The high-quality open source epilepsy datasets are divided into five subsetss of ictal scalp EEG signals {F, N, O, Z, and S}, which are collected by 25 subjects. Among them, both subsets {O} and {Z} are provided by five healthy volunteers, with eyes open and closed, respectively, and were collected from scalp surface EEG. Subsets {FN} and {S} contain EEGs from epileptic patients, of which subsets {FN} were, respectively, recorded in seizure-free intervals from five patients and subset {S} includes seizure activity recorded from all intracranial sites. All EEG signals were extracted by the 128-channel amplifier system with an average common reference. Each subset contained 100 samples of EEG signals from 5 subjects. The raw EEG signals were recorded using an international standard 10–20 system with a 173.61 Hz sampling frequency using 12-bit resolution. The age of the subjects ranged from 19 to 60 years. They were all right-handed, and the locations of the epileptogenic foci for each subject were identified by experienced neurologists or epileptologists.

The five EEG datasets {F, N, O, Z, S} were subjected to standard normalization procedures and were combined into three types: {S}, {FN}, and {OZ}, according to the level of disease, which is continuous ictal epilepsy patients, intermittent epilepsy patients, or healthy volunteers, respectively. More detailed information about the five EEG datasets {F, N, O, Z, S} is provided in Table 1.

The major goal of the system proposed in this paper is to classify the existing EEG signals into the three types: {FN}, {OZ}, and {S}. First, the raw EEG signals were preprocessed using the open source toolbox, EEGlab, running under MATLAB. This involves several steps, including Butterworth filtering, removal of artifacts, baseline corrections, and separation of the data into segments [43].

### 3.2. Feature Extraction Using the Symlet Wavelet

The Discrete Wavelet Transform (DWT) analysis was able to accommodate the properties of non-stationary signals. The effective frequency range obtained after band pass filtering was 0 to 50 Hz. The DWT decomposes the EEG scalp signals at time *t*, expressed as:

(1) $$s(t)={\left(s_{1}(t),s_{2}(t),\cdots ,s_{n}(t)\right)}^{T}=\left(\begin{array}{ccc} s_{11} & \cdots & s_{1p}\\ \vdots & \ddots & \vdots \\ s_{n1} & \cdots & s_{np} \end{array}\right),(i=1,2,\cdots ,n)$$

where s(t) represents the n×p EEG signals, k=1,2,⋯,n/2, $s_{n}(t)$ is the nth channel of EEG, and *p* is the number of scales, into the wavelet, $\omega_{p,k}(t)$, and scaling, $\varphi_{p,k}(t)$, functions defined, respectively, as follows:

(2) $$\omega_{p,k}(t)=2^{-p/2}\omega (2^{-p}t-k)$$

(3) $$\varphi_{p,k}(t)=2^{-p/2}\varphi (2^{-p}t-k),p,k\in Z$$

**Figure 2:**
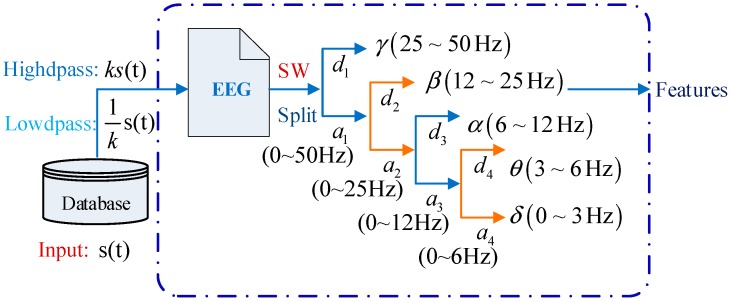
Frequency bands of epilepsy EEGs extracted using wavelet decomposition.

The DWT results in a hierarchy of decompositions at four levels, as illustrated in Figure 2. At each level, p, the approximation can be calculated using (4):

(4) $$D_{p}(t)=\sum_{k\in Z}C(p,k)\omega_{p,k}(t)$$

Here, $C(p,k)=\int_{-\infty }^{+\infty }s(t)\omega_{p,k}(t)dt$ denotes the wavelet coefficients.

EEG signal s(t) can be defined as the sum of all detail coefficients [44]. At level P, the detail coefficients with exponents, p≤P, represent fine details, whereas those with p>P represent the coarse details. The coarse detail is also called the approximation, $A_{p}(t)$, of signal, s(t), and is defined as $A_{p}(t)=\sum_{p>P}D_{p}(t)$. As the level, P, increases, the resolution, $2^{-P}$, decreases and $A_{p}(t)$ contains only the low frequency features of the EEG.

In this study, the Symlet wavelet was used to extract a specific frequency band from EEG signals. The Symlet wavelet is an improvement on the Daubechies wavelet, which addresses the disadvantage of the approximate asymmetry present within the Daubechies wavelet. The support range and vanishing moment of the Symlet wavelet are 2N-1 and N, respectively. The Symlet wavelet basis has better regularity than Daubechies, and this can reduce the phase distortion in the analysis and reconstruction of non-stationary signals, such as EEGs [45].

**Figure A1:**
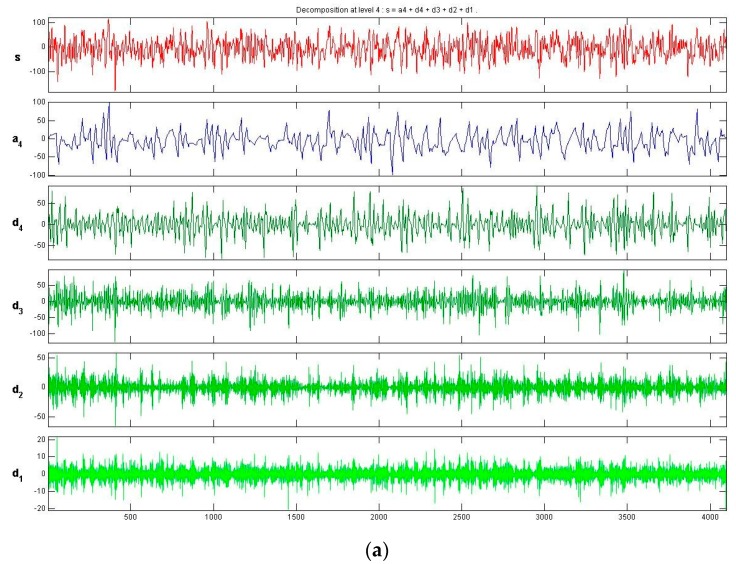

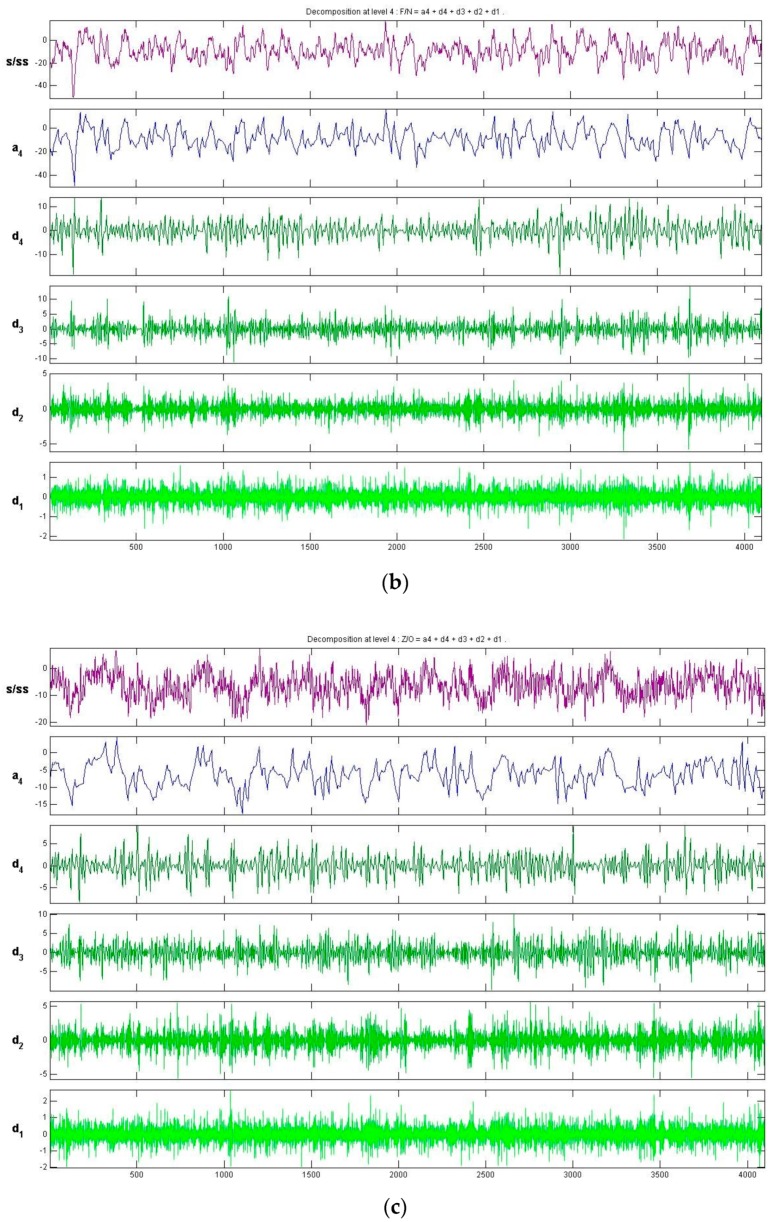
(**a**) Raw {S} data and corresponding wavelet decomposition; (**b**) raw {FN} data and corresponding wavelet decomposition; and (**c**) raw {OZ} data and corresponding wavelet decomposition.

Figure A1a–c of Appendix A show the SW visual decomposition process for the continuous epilepsy {S}, intermittent epilepsy {FN}, and healthy subject {OZ} EEGs. The raw EEGs shown in the first column are divided into several feature segments according to the frequency ranges of γ (25–50 Hz), β (12–25 Hz), α (6–12 Hz), θ (3–6 Hz), and δ (0–3 Hz). In the first decomposition process, the detail coefficient, $d_{1}$, and approximation coefficient, $a_{1}$, are generated. Next, $a_{1}$ is injected into the SW to generate detail coefficient, $d_{2}$, and approximation coefficient, $a_{2}$. The other wavelet coefficients are obtained in a similar way. The decompositions for {S}, {FN}, and {OZ} of the EEG datasets are shown in rows 2 to 6 of Figure A1a–c.

The mean and standard deviations show the density of the center and range of possible EEG signal values, and are respectively defined as follows. In practice, it is necessary to extract statistical information from the time–frequency features of EEG signals, which can be understood as continuous random variables [46]:

(5) $$\mathrm{mean}=\int_{-\infty }^{+\infty }s(t)P(s(t))ds(t)$$

(6) $$ST=\int_{-\infty }^{+\infty }{\left(s(t)-\omega \right)}^{2}P(s(t))ds(t)$$

The absolute value of the EEG is taken to avoid negative energy. To ensure the credibility of the test results, arithmetic average processing was performed for the above three groups of data and they were compressed into single column matrices. The energy mean, number of cases, and variance of the {FN}, {OZ}, and {S} EEGs are shown in Table 2. EEGs in {S} have the largest standard deviation and highest mean energy.

When extracting features, it is also necessary to use PCA after the Symlet wavelet to obtain low-dimensional features. PCA ensures the information is as relevant as possible. It constructs a new feature subspace from the information derived from the existing features. This procedure reduces the computational load on the recognition system and increases the computational efficiency.

### 3.3. Classifier Implementation

There are many machine learning pattern classifiers that could be used to classify EEG data, and it is difficult to choose the most suitable one for the analysis of multi-class epilepsy EEG data. In the following section, we discuss the most widely used SVM and RF classifiers as well as the GBM classifier proposed in this paper.

#### 3.3.1. Gradient Boosting Machine

In this study, the gradient boosting machine algorithm is used to train the classifier. The GBM is a method for the gradual enhancement or improvement of error. It was designed by Friedman [47], who considered estimation of the functional dependence, y=η(s(t)). The loss function describes the level of robustness of the classification model. The best method to improve the classification model is to make the loss function descend at its gradient direction [48,49]. In this study, the GBM was used to train the classifier. We expressed the EEG training by $M=\left\{m_{p}\in R^{k},p=1,2,\cdots ,N\right\}$, where N is the number of EEG of the dataset, and $m_{p}$ expresses the feature vector of the *p*th EEG. In the model of the construction process, loss function, ψ(y,η), is minimized as follows:

(7) $$\overset{\wedge }{\eta }(s(t))=\overset{\wedge }{y}=\arg\min\psi \left(y,\eta \right)$$

The function estimate, $\overset{\wedge }{y}=\sum_{i=1}^{M}{\overset{\wedge }{y}}_{i}$, is parametrized with ${\overset{\wedge }{y}}_{i}$, which is defined as a boost. We created a greedy strategy that estimates ${\overset{\wedge }{y}}_{k}={\overset{\wedge }{y}}_{k-1}+\Delta_{k}\cdot \xi \left(\bar{s(t)},\theta_{k}\right)$ at each recursion, where $\xi \left(\bar{s(t_{i})},\theta \right)$ is called the base learner; that is, a decision tree. The function is built as follows:

(8) $$\left(\Delta_{k},\theta_{k}\right)=\arg\min_{\Delta ,\theta }\sum_{i=1}^{N}\psi \left(y^{\left(i\right)},\overset{\wedge }{\eta_{k-1}}\right)+\Delta \cdot \xi \left(\bar{s(t_{i})},\theta \right)$$

While this optimization problem is hard for a general loss function and the base learners, Friedman suggested a new function, $\xi \left(\bar{s(t)},\theta \right)$, which is the function that is the closest to being parallel to the negative gradient along the observed data, whereby the optimization task becomes a classic least-squares minimization. Table 3 lists the pseudo code of GBM.

#### 3.3.2. Parameter Optimization and CV

• Parameter optimization

The GBM identification algorithm generates decision tree and boosting parameters during the training process. Although the GBM classifier does not result in much over-fitting as the decision tree grows, the high learning rate still causes over-fitting of the classification model. If we reduce the learning rate and increase the decision tree blindly, the calculations can be very expensive and take a long time to run. This paper proposes an improved GSO to optimize the parameters of the GBM classification model to improve the classification performance of the GBM classifier. The specific steps are as follows.

**Figure 3:**
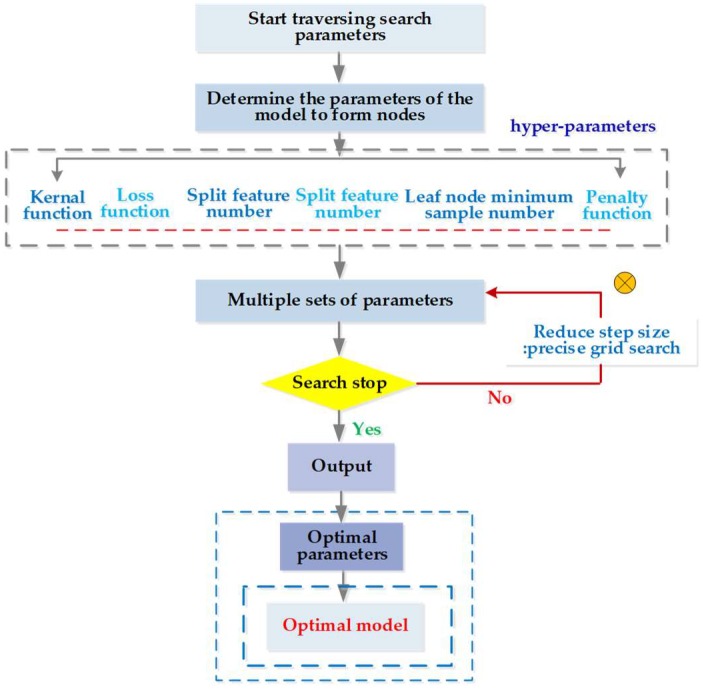
Parameters optimization flow in the grid search optimized algorithm.

First, we used a long-distance step size for a rough search over a large range. Second, the mesh was built on the coordinate system, with its mesh nodes being the corresponding parameter pairs of the decision trees and boosting. The optimal parameters and recognition accuracy were output when there was a set of parameters that met the requirements; we selected the parameter with the smallest penalty parameter as a more selective object when multiple sets of parameters met the requirements. Next, a second accurate search was performed in small steps on the set of parameters: The above steps were repeated with the step set to 0.1 to find the global optimal hyper-parameters. A flowchart of this proposed parameter optimization is shown in Figure 3.

Generally, the default value for the learning rate is 0.1; however, for different problems, values between 0.05 and 0.2 can determine the optimal number of decision trees at the current learning rate. In this study, the optimal learning rates determined by the GSO algorithm was found to be 0.06.

• 10-fold CV

The automatic seizure detection systems of Guo et al. [50], Nicolaou et al. [51], Samiee et al. [52], and Yuanfa Wang et al. [37] did not use CV, whereas Qu et al. used the default 5-fold CV (32). To reduce the influence of the selected training and testing data on the model verification, 10-fold CV was used. In this process, the training data were randomly divided into 10 subsets without repetition. The other residual subsets were used to train the EEG classifier on data corresponding to different levels of epileptic seizures. The division process is expressed as the following formula (9):

(9) $$\left\{V_{1},V_{2},\cdots ,V_{k}\right\},\left(V_{i}\cap V_{j}=\emptyset \right)$$

This process was repeated 10 times to obtain 10 accuracy measurements. After 10 operations, the average was used as the final CV error, $CVe=\frac{1}{10}\sum_{q=1}^{10}e_{q}$, for selecting the classifier, where $e_{q}=\frac{1}{m}\sum_{n=1}^{m}{\left(\overset{\wedge }{y_{n}}-y_{n}\right)}^{2}$ is the average error of the qth test set and m is the number of samples in the qth test set.

**Figure 4:**
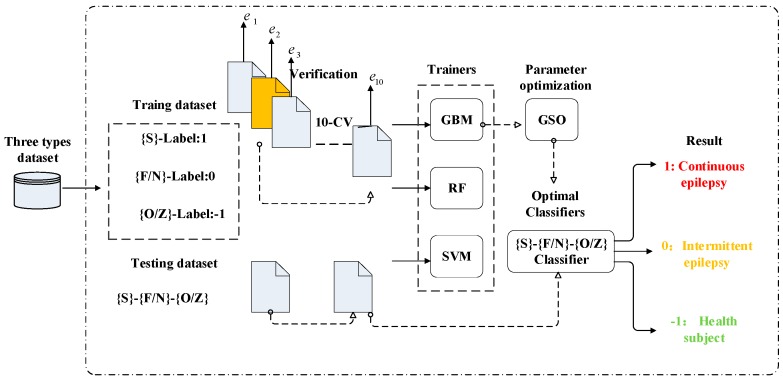
Classification implementation.

The architectures of the three types of classifiers used for epilepsy detection are shown in Figure 4. The training data and their corresponding labels were included in the dataset for each category. The EEGs for {S}, {FN}, and {OZ} were decomposed into five frequency sub-bands using four levels of SW. The mean and standard deviation values of the wavelet coefficients were then calculated to create a 10-dimensional feature vector. The training sets for {S}, {FN}, and {OZ} were labeled with “1”, “0”, and “−1”, respectively. The 10-dimensional feature vector and pre-trained SVM, RF, and GBM classifiers act in the feature recognition module of the scheme. During the training process, the GSO searches of the optimal values for the generated parameters.

## 4. Experimental Results and Discussion

The experiments were performed on an Acer PC with a 2.8 GHz Intel Core i5-6200U CPU, 8 GB of low voltage memory, 1 TB of storage, and a 64-bit operating system.

### 4.1. Multiple Performance Evaluation and Results Comparison

After the design of the proposed system is complete, it is essential to conduct an evaluation that employs multiple performance indices. We evaluated the model using not just accuracy, but also multiple evaluation indices commonly used in machine learning. Furthermore, these results can be used to adjust the model so that it can achieve a higher accuracy. The main evaluation indices consist of CM, the ROC, and AUC. These indices enable a deeper analysis of the performance of a classification model from the perspective of classification errors, which is more important in medical diagnosis detection. The performance indicators’ accuracy, sensitivity, and specificity are defined in Table 4 for the three-class classification of epilepsy EEGs.

Table 5 summarizes the processing results of the Bonn University data over recent years, including the techniques used, number of classification levels, and results of multiple-index evaluations. As listed in Table 5, almost all researchers have classified the data into two-class, {Z}-{S}, {O}-{S}, {N}-{S}, {F}-{S}, {OZ}-{S}, {NF}-{S}, {OZ}-{NF}, or {FNOZ}-{S} [29,51,53,54]. The exception is Wang et al., who conducted three-category {FN}-{OZ}-{S} classification in 2017, and achieved an accuracy rate of 93.9% [37]. Our proposed method achieved better results on the three-category problem, with an accuracy of 96.5%. Many different machine learning performance indicators have been evaluated for the SM-GBM-GSO approach, as shown in Table 5. These results lead us to infer that our proposed approach exhibits potential for automated three-class classification of epilepsy EEGs.

**Figure 5:**
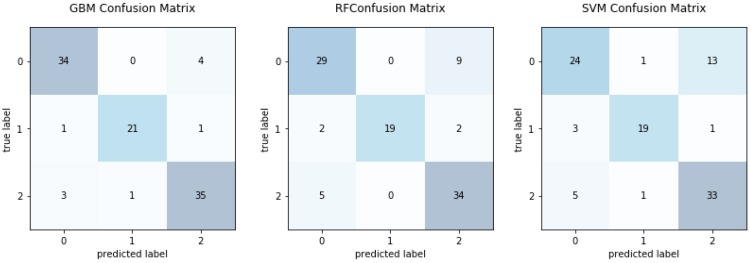
Confusion matrices comparing the results of gradient boosting machine, random forest and support vector machine with grid search optimizer on {FN}-{OZ}-{S} classification.

Furthermore, we compared the CMs for the three EEG dataset categories, {S}, {FN}, and {OZ}, labeled by the GBM, RF, and SVM classifiers in Figure 5. The horizontal and vertical directions of a CM indicate the real and predicted classes, respectively. The GBM classifier achieved higher numbers of correct classifications than the RF and SVM classifiers. There were two main types of classification error. The first type occurred when the serious disease {S} class was mislabeled as {OZ} (with a probability of 1%). The second type occurred when class {S} was misclassified as {FN} (also with a probability of 1%). The proposed method not only has a high rate of true positives and true negatives, as can be seen on the main diagonal line, but also avoids errors from false positives and true negatives, as represented by the off-diagonal line.

**Figure 6:**
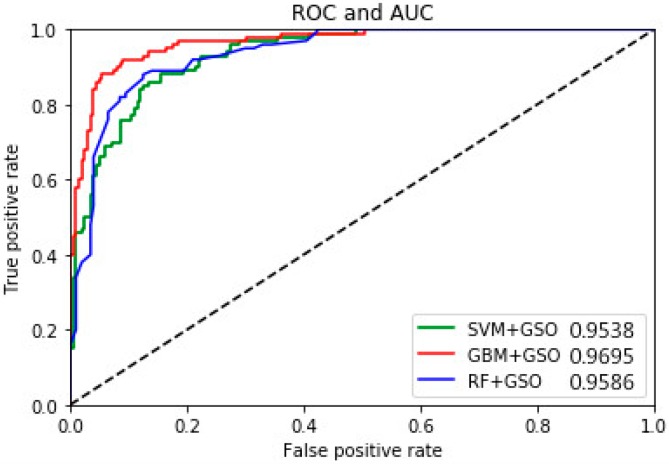
Comparison of receiver operating characteristics for the three-class classification.

With the technological developments in machine learning over recent years, the identification accuracy and confusion matrix can be considered insufficient to judge the accuracy of a classification. We can construct a classifier with high accuracy or recall, but it is difficult to ensure both at the same time. Therefore, we used the ROC and AUC [57] to assess the performance of the classifiers [58]. To allow an ROC curve to be drawn, the classifier must provide a confidence value that is judged as positive or negative for each sample. If the ROC curve falls above the diagonal, this indicates the classification model has predictive ability, and conversely, there is no predictive ability. The ideal situation is that the ROC curve coincides with the y-axis, that is, the prediction ability is 100%. The AUC defines a natural measure for overall performance assessment of a classifier based on the ROC. Li et al. also used the AUC index for their results on the same dataset, but their values of 0.66–0.87, as shown in Table 6, are not very satisfactory [54]. Figure 6 summarizes the AUC comparisons between the proposed GBM, RF, and SVM identifiers with GSO using subsets {FN}-{OZZ}-{S}, with values of 0.9695, 0.9586, and 0.9538, respectively. In medical detection, a high true-positive rate is more desirable for a fixed lower-false positive rate. By definition, we consider the higher true-positive value to be the better one.

**Figure 7:**
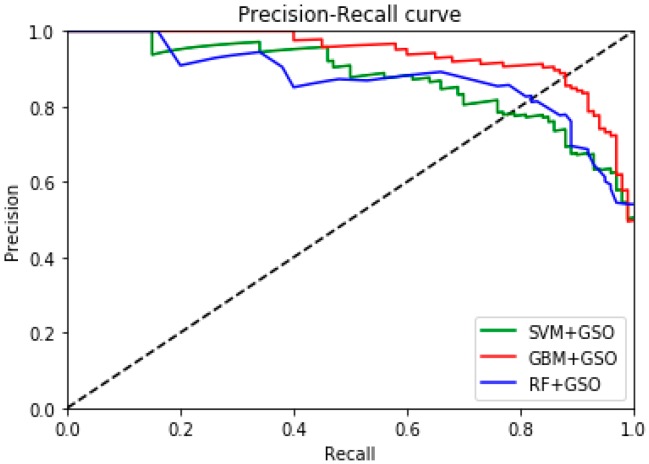
Comparison of the precision–recall curves space for the three-class classification.

The precision recall curve (PRC) has a wide range of applications in the field of classification and retrieval; it represents the relationship between precision and recall. The precision values of the vertical axis represent the correct predictions as the ratio of positive samples to all positive samples, while the recall of the horizontal axis represents the correctly predicted ratio of positive samples to true samples. When the precision and recall are high, we can be assured that the classification performance is good. It can be seen in Figure 7 that the GBM-GSO classifier has the best performance in the three-class classification according to the multiple indicators of ACC, CM, ROC, AUC, and PRC.

### 4.2. Comparative Analysis of Classifiers

All in all, one of the aims of the method proposed in this paper is to maintain robustness while solving multi-class classification, thereby ensuring recognition accuracy. SVMs are widely used for classifying EEGs, and RFs can achieve excellent performance in pattern recognition. We compared the proposed GBM method with an SVM and RF, discussing them with respect to three main aspects: Multi-class classification; the sensitivity of parameter selection; and the generalization ability. The conclusions obtained are summarized in Table 6, which can provide valuable references for other researchers using pattern recognition systems.

• Multi-class classification problem

The SVM was initially used to separate EEG data into two types by finding the optimal hyperplane, *ω^T^* + *b*. The idea of maximizing the classification margin is the core of the SVM method [59]. In the application of data mining, it is generally necessary to solve a classification problem with multiple classes in practice. This can only be solved by constructing a combination of several two-class classifiers. Although the multi-class classification problem can be solved this way, it is cumbersome and does not guarantee good precision [37]. In contrast, RF and GBM are decision tree models based on integration ideas, and they are better suited for solving multi-class classification problems.

• Sensitivity of parameter selection

The performance of an SVM classifier depends mainly on the selection of the kernel function; therefore, a practical problem is how to choose the appropriate kernel function. At present, a more mature approach is to artificially choose the kernel function and its parameters based on experience plus an element of randomness. Kernel functions should have different forms and parameters for different problem areas, and so domain knowledge should be introduced when making the selection. Currently, there is no good way to solve the problem of kernel function selection.

• Generalization ability

The main characteristic of the RF classifier [60] is the selection of features using the principle of Gini index minimization. Because of the random selection of samples and features, it is not easy to over-fit the data. In the bagging step of RF, a tree is grown to obtain an average predictive power across all decision trees using a parallel boosting method. Each tree is constructed on a sample of raw data and the results of the trees are voted on to achieve the result without further optimization of the training results of different trees.

The essential difference between GBM and RF is that each tree in GBM learns the residuals of all previous tree conclusions. The residual is the true value minus the predicted value. GBM is superior to RF in that it is not based on decision trees built in parallel. The construction of a GBM classifier involves moving along the direction in which the gradient drops the fastest. The gradient generates a completely new decision tree at each iteration. To make up for the lack of an original recognition system, the partial derivative of the loss function at each training sample point is used to construct a weak learner. Therefore, the GBM classification system has stronger generalizing ability and better adaptability to new data than the RF and SVM techniques.

The above comparative analysis indicates that the GBM classifier is the most suitable for the three-class classification problem of epilepsy in EEGs.

### 4.3. Contribution and Advantages of the Proposed System

Analyzing epilepsy EEGs from a computer recognition point of view can help promote our understanding about the state of an illness. We believe that our machine learning approach has the following contributions and advantages for the classification of epilepsy EEGs.

(a) It not only enables representation of the core time–frequency information of EEGs through wavelet transforms, but also extracts key statistical information. The statistical information of time–frequency features are used as recognition features, and these features reflect the overall characteristics of the data. Simultaneously, a PCA is adopted to reduce the dimensionality of the data. Thus, the proposed method reduces the amount of hardware calculation under the premise of guaranteeing the accuracy of the classifier.
(b) The proposed GBM recognition system was highly parallelized to improve operational efficiency. Another advantage is that it can process large-scale data. However, the recognition system generates many parameters in the course of the training process, and it can be difficult to determine the optimal parameters by manual tuning. This paper proposes a GSO to optimize these parameters and determine the best recognition system filtering parameters by repeatedly varying the step size. To prevent over-fitting in the GBM training process, we adopted a 10-fold CV strategy, which ensures that the optimized system is more robust.

The integrated SW-GBM-GSO methods classify three classes: Healthy subjects, intermittent epilepsy patients, and continuous ictal epilepsy patients. We used multiple performance indicators to evaluate and verify the classification system. In addition to classification accuracy, ACC, CM, PRC, ROC, and the AUC were measured. These indicators enable a more thorough and clearer analysis of the error rate resulting from misclassification. This strategy is pivotal in medical screening.

## 5. Conclusions

The use of EEG signals has changed the method of monitoring epileptic seizures. In this study, the SW-GBM-GSO was proposed for an auxiliary medical diagnostic system ofepilepsy EEGs. The proposed method performed well at the classification of healthy subjects, intermittent epilepsy, and continuous ictal epilepsy. In this system, Symlet wavelets are used to decompose the EEG data into five time–frequency sub-bands and the mean and standard deviation of statistical features were also calculated. Subsequently, a modified GSO is used to search for the optimal parameters using a variable-step method. The use of 10-fold CV avoids overfitting of the classifier. We compared GBM with SVM and RF for the classification of EEG data. Considering that most other schemes have only been concerned with classification accuracy, we focused on multiple indicators to determine the misclassification factors. These indicators are essential in medical screening. According to the experimental results and multiple evaluation indicators, we conclude that the proposed Symlet wavelet processing, a GBM, and a GSO together obtain the highest performance in the three-class classification.

In the future, we intend to optimize our detection approach to achieve higher recognition rates for multiple levels of epileptic seizure. We also hope to transfer the technology out of the laboratory and plan to develop a smart mobile phone application to assist medical diagnosis of an epilepsy patient. The EEG signals would be transmitted to a mobile terminal through a wireless sensor network [61,62]. The scheme could assist medical diagnosis and be used to alert medical professionals to an epileptic occurrence. It should be especially useful for people or infants who suffer paroxysmal epilepsy and who could be monitored at home in the evening.

## Figures and Tables

**Table 1:** Dataset description.

Data Sources	Parameter Description	Dataset Category	Subject Condition	Epileptogenic Foci	Electrode Collection Area	Number of Samples
Bonn University	5 groups 173.6 Hz. 23.6 s. 4096 data points.	{OZ}	Healthy volunteers	Scalp surface	All brain areas	200
		{FN}	Intermittent epilepsy	Intracranial site	Lesion outside inside area	200
		{S}	Continuous ictal epilepsy	Intracranial site	Intra-lesional area	100

**Table 2:** Statistical features of the dataset.

Datasets	{FN}	{OZ}	{S}
Mean	−5.94	−6.31	−4.74
Number of cases	4097	4097	4097
Standard deviation	13.10	4.56	38.55

**Table 3:** Gradient boosting machine classifier in pseudocode.

**ALGORITHM**: Gradient Boosting Machine (GBM)
**Data:**n observed data features {T-F features, statistical features $\bar{s(t_{i})}$}
**Process:** Calculate loss function ψ(y,η) and base-learner classifier $\xi \left(\bar{s(t)},\theta \right)$ to number of iterations *M*.
Build predicted classifier $\overset{\wedge }{\eta }(s(t))$ for $\bar{s(t)}$. Initialize ${\overset{\wedge }{\eta }}_{0}=\arg\underset{\Delta_{k}}{\min}\sum_{i=1}^{N}\psi \left(\bar{s(t_{i})},\Delta_{k}\right)$; for m∈{1,2,⋯,M} Compute the negative gradient $\zeta_{k}(s(t))$; Fit a new base-learner function $\xi \left(\bar{s(t)},\theta_{k}\right)$; Find the best gradient descent step-size $\Delta_{k}$ to obtain a tree classifier: $$\Delta_{k}=\arg\min_{\Delta \theta }\sum_{i=1}^{N}\psi \left(y^{\left(i\right)},\overset{\wedge }{\eta_{k-1}(s(t_{i}))}\right)+\Delta \cdot \xi \left(\bar{s(t_{i})},\theta_{k}\right)$$ Update function $\eta_{k}=\Delta_{k}\zeta_{k}(s(t))$ and the GBM classifier $\eta \left(\bar{s(t_{i})}\right)=\eta_{k}+\eta_{k-1}$;
**end for**;
**return**$\eta \left(\bar{s(t_{i})}\right)$;

**Table 4:** Definition of the performance classification multiple-indices used in the experiments. Parameters, $A_{ij}$ (i=j), are the probability of correct classification for sub-datasets {i}. Similarly, $A_{ij}$ (i≠j) represents the incorrect classification probability. Parameters, $A_{i}=\sum_{i=1}^{3}A_{1i}$, are the sum of all classification rates of sub-datasets, {i} (i,j=1,2,3).

Test/Real Type	{OZ}	{FN}	{S}	Sensitivity (SEN)	Specificity (SPE)	Accuracy (ACC)
{OZ}	$A_{11}$	$A_{12}$	$A_{13}$	$\frac{A_{11}}{A_{1}}$	$\frac{A_{22}+A_{23}+A_{32}+A_{33}}{A_{2}+A_{3}}$	$\frac{A_{11}+A_{22}+A_{33}}{\mathrm{All}}$
{FN}	$A_{21}$	$A_{22}$	$A_{23}$	$\frac{A_{22}}{A_{2}}$	$\frac{A_{11}+A_{13}+A_{31}+A_{33}}{A_{1}+A_{3}}$	
{S}	$A_{31}$	$A_{32}$	$A_{33}$	$\frac{A_{33}}{A_{3}}$	$\frac{A_{11}+A_{12}+A_{21}+A_{22}}{A_{1}+A_{2}}$	

**Table 5:** Comparison of results of the proposed method with those of existing methods for accuracy, area under the curve, receiver operating characteristic, confusion matric and precision–recall curve of for the two- and three-level-class classifications on the Bonn University data.

Authors	Techniques	10-Fold CV	Dataset	ACC (%)	AUC	CM/PRC
Guo et al. (2010) [55]	DWT and line length, ANN	No	{Z}-{S} {FNOZ}-{S}	100 97.7	No	No
Gandhi et al. (2011) [53]	DWT, energy and std, SVM, NN	Yes	{FNOZ}-{S}	95.4	No	No
Nicolaou et al. (2012) [51]	Permutation entropy, SVM	No	{Z}-{S} {O}-{S} {N}-{S} {F}-{S} {FNOZ}-{S}	93.5 82.8 88.0 79.94 86.1	No	No
Shafiul Alam and Bhuiyan et al. (2013) [56]	EMD, higher order moments, ANN	No	{O}-{S} {F}-{S} **{FN}-{OZ}-{S}**	100 100 **80**	No	No
Samiee et al. (2015) [52]	STFT Spectral coefficients with their statistical, values, Bayes, LR, SVM, KNN, and ANN	No	{Z}-{S} {O}-{S} {N}-{S} {F}-{S} {FNOZ}-{S}	99.8 99.3 98.5 94.9 98.1	No	No
Swami et al. (2016) [53]	DTCWT, energy and std, Shannon entropy features, RNN	Yes	{Z}-{S} {O}-{S} {N}-{S} {F}-{S} {OZ}-{S} {NF}-{S} {FNOZ}-{S}	100 98.89 98.72 93.3 99.1 95.1 95.2	No	No
Li et al. (2016) [54]	Distribution entropy and sample entropy Statistical analysis	No	for sample entropy distribution entropy for short length data	mean	Yes 2-class classification 0.93–0.97 0.66–0.87	No
Manish et al. (2017) [29]	ATFFWT and FD, LS-SVM	Yes	{Z}-{S} {O}-{S} {N}-{S} {F}-{S} {OZ}-{S} {NF}-{S} {OZ}-{NF} {FNOZ}-{S}	100 100 99 98.5 100 98.6 92.5 99.2	No	No
Wang et al. (2017) [37]	DWT, SVM	No	**{FN}-{OZ}-{S}**	**93.9**	No	No
**This work**	Symlets wavelets, statistical mean energy std and PCA, GBM-GSO, RF, SVM	Yes	{Z}-{S} {O}-{S} {N}-{S} {F}-{S} {OZ}-{S} {NF}-{S} {OZ}-{NF} {FNOZ}-{S} **{FN}-{OZ}-{S}**	100 100 98.4 98.1 100 98.1 93.2 98.4 **96.5**	Yes 3-class classification GBM –GSO 0.9695 RF –GSO 0.9586 SVM –GSO 0.9538	Yes

**Table 6:** Performance comparisons between gradient boosting machine, support vector machine and random forest.

	GBM	SVM	RF
Multi-class classification ability	★★★	★	★★★
Sensitivity of parameter selection	★	★★	★★
Generalization ability	★★★	★★	★★
Strong: ★★★ Moderate: ★★ Weak: ★
